# Effects of traction therapy on atlantoaxial joint dislocation-induced cervical vertigo

**DOI:** 10.1590/1414-431X2022e11777

**Published:** 2022-02-28

**Authors:** Genghui Cai, Dabin Zhu, Jieyun Chen, Xiuyao Lin, Ri Chen

**Affiliations:** 1 Department of Rehabilitation, Quanzhou First Hospital Affiliated to Fujian Medical University, Quanzhou, Fujian, China; 2 Department of Radiology, Quanzhou First Hospital Affiliated to Fujian Medical University, Quanzhou, Fujian, China

**Keywords:** Atlantoaxial joint dislocation, Cervical vertigo, Traction therapy

## Abstract

Cervical vertigo is a common complication of atlantoaxial joint dislocation. However, there is no consensus on the effects of different therapies on the recovery of the patients suffering cervical vertigo. The objective of this randomized controlled trial was to investigate the effect of traction therapy on reducing cervical vertigo induced by atlantoaxial joint dislocation. A total of 96 patients were randomized to receive traction therapy or traditional therapy for two weeks. The overall clinical efficacy was measured based on the 30-point cervical vertigo symptom and function evaluation form. The therapeutic effects were also evaluated based on lateral atlantodental space (LADS), vertigo scale, neck and shoulder pain scale, headache scale, daily life and work scale, psychosocial adaptation scale, and quality of life. Compared with the traditional therapy group, the traction group demonstrated markedly higher overall clinical efficacy (P=0.038). Both the traction therapy group and the traditional therapy group showed significant decrease in LADS (P<0.001), but the traction therapy group had a greater reduction of LAD compared with the traditional group (P<0.01). Traction therapy consistently led to significantly greater relief of cervical vertigo symptoms, including dizziness, neck and shoulder pain, headache, inconvenience in daily living and work activities, impaired psychosocial adaptation, while improving quality of life. The efficacy of traction therapy for cervical vertigo surpasses that of traditional therapy, suggesting that traction therapy is potentially more clinically useful in treating these patients.

## Introduction

Atlantoaxial joint dislocation (AJD) is a common consequence of traumatic dislocation of the upper cervical spine or non-traumatic causes such as congenital causes, inflammation of the nasopharynx, and rheumatoid arthritis (1,2). Therapy of AJD consists mainly of conservative manual therapy and invasive surgical therapy (3). In manual therapy, manipulation of cervical bones and muscles is performed to bring the cervical spine into the neutral position using a rotational movement, which is commonly practiced by both western chiropractors and traditional Chinese herbalists (4). However, cervical manipulation sometimes triggers a vertiginous attack that stems from decreased blood perfusion, leading to ischemic change in the cerebellum, inner ears, or brainstem (5-7). Cervical vertigo is manifested as imbalance, light-headedness, or unsteadiness. Some patients with cervical vertigo experience illusion of movement, spinning or whirling, which lead to a certain level of disability and deteriorates quality of life (8). Unfortunately, insufficient attention has been devoted to cervical vertigo in choosing the therapies for AJD. Therefore, there is an urgent need to establish effective clinical treatment methods of AJD that minimizes cervical vertigo.

The traction method for treatment of AJD is a novel technique adopted that is increasingly used in clinical practice (9-11). This method is thought to reduce cervical vertigo compared with the traditional method. The traction method has been traditionally applied to treat cervical vertigo by other spinal diseases (12-14). The benefits of traction have been shown to be an increase in blood flow (15) and a decrease in pain (16-18). However, the clinical benefit of traction therapy in alleviating cervical vertigo induced by AJD has not been fully supported due to the lack of randomized controlled clinical trials.

The aim of the study was to conduct a randomized controlled clinical trial to compare the effects of traction therapy and traditional therapy in reducing cervical vertigo, along with clinical symptoms and quality of life associated with cervical vertigo. The results provided by the study could shed light on the clinical benefit of traction therapy in reducing cervical vertigo as a treatment for AJD.

## Material and Methods

### Enrollment

This study was approved by the Ethic Committee of Quanzhou First Hospital Affiliated to Fujian Medical University (registration number ChiCTR2100051942). The subjects of this study were recruited from the Rehabilitation Medicine, Orthopedics, Neurology Outpatient Clinics, and wards of the First Hospital of Quanzhou City. Inclusion criteria were: 1) diagnosis of atlantoaxial disorder; 2) age between 18 and 60 years, regardless of gender; 3) dizziness caused by diseases such as cardiovascular, otolaryngology, neurology, internal medicine, orthopedics, etc.; and 4) voluntarily participation in the trial and signing the informed consent. The main symptoms used as diagnostic criteria were dizziness, migraine, neck pain, and posterior occipital pain, accompanied by symptoms of autonomic dysfunction. In severe cases, visual rotation, nausea, tinnitus, insomnia, and scalp numbness may be seen. Anatomical signs are C2 spinous process positioned to one side, swelling and pain in the tissues surrounding the vertebra, head and face skewed to the side, and limited movement of the cervical spine. These signs are aggravated when turning the head or changing position, and the neck flexion test is positive. Lateral radiographs show shallow or straightened cervical physiological curvature, normal radiographs show varying degrees of vertebral body rotation below C3, suggesting structural instability of the upper cervical segment. Open-mouth radiographs show unequal distance between atlas and teeth, with a variance of 14 mm and no fracture.

Exclusion criteria were: 1) patients with cervical spondylotic radiculopathy and severe spinal canal stenosis; 2) patients with history of spinal surgery or severe spine trauma; 3) known congenital variation or deformity of unstable cervical spine structure; 4) spinal infections, fractures, tumors, tuberculosis, severe spinal deformities, severe osteoporosis, ankylosing spondylitis, deformity osteitis, and other diseases seen in imaging studies; 5) severe primary diseases of the endocrine system, primary cardiovascular disease, autoimmune disease, tumor, or psychosis; 6) dizziness caused by otogenic (vestibular), ocular, cerebral (central systemic), drug-induced, poisoning, and other causes; 7) being pregnant or recently preparing for pregnancy and breastfeeding women; and 8) patients who had received or were receiving other treatments within the past 3 months, which affect the efficacy of the tested treatment.

Patients were randomized using a web-based system (Institute of Basic Research in Clinical Medicine, China Academy of Chinese Medical Science) to receive traction therapy or traditional therapy. Patients and the investigators who analyzed the data were blind to treatments.

### Treatment methods

#### Traction therapy

**Figure 1:**
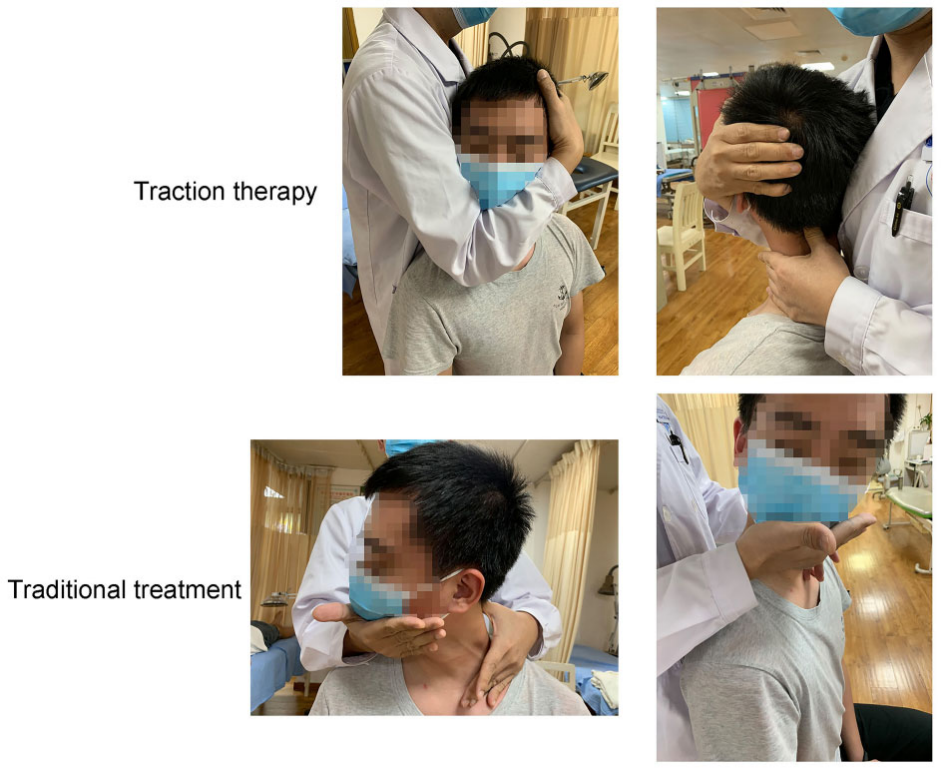
Procedures of traction therapy and traditional treatment.

The procedures for traction therapy are shown in Figure 1. The patient adopted a sitting position. To treat patients with the left deviation of the C2 spinous process, for example, the surgeon stood behind the patient and pressed the left edge of the C2 spinous process with the thumb of the left hand, with the right elbow flexed to clamp the patient's mandible. The surgeon placed the palm around the ears. Traction was conducted along the longitudinal axis of the spine for about 1 min. With traction, the cervical spine was slowly rotated to the right to the C2 spinous process protruding position. Then a controlled, slowing increasing movement was enforced using the left thumb to push the C2 spinous process to the right. When a popping sound and feeling at the C2 spinous process was achieved, the procedure was complete.

For patients with forward incline, the patients were placed in supine position with a pillow on the neck. The surgeon sat at the top side of the patient's head, supporting both side of the patient's head using hands with the middle finger on both sides of the C2 spinous process. Cervical traction along the longitudinal axis of the spine was conducted for 2-3 min, and then the patient was instructed to take a deep breath. At the end of exhalation, intermittent upward force was applied to the patient's head. The surgeon would look for a popping sound or a sense of movement at the C2 spinous process.

#### Traditional therapy

The steps of this therapy are shown in Figure 1. The traditional cervical spine rotation method was used. The patient was in a sitting position with the neck leaning forward by 10 to 15°. The physician stood behind the patient and pressed the cervical spinal process or lateral process of the patient's cervical spine with one hand, while holding the chin with the other hand to slowly rotate the head. When the rotation found resistance, the physician then made a controlled larger rotation with greater force. At the same time, the spinous process was pushed hard to the opposite side with coordinated movement by the two hands. When a popping sound and feeling was achieved at the C2 spinous process, the manipulation was considered successful.

All patients in the two groups were treated by the same physician. According to the patient's symptoms, manipulations were performed 3 to 4 times a week. Other treatments were mainly muscle message therapies. Each treatment lasted about 15-20 min, and each treatment course was 14 days. The therapeutic effect was observed after a treatment course.

### Evaluation of treatment efficacy

The primary outcomes of our trial were changes in the lateral atlanto-distal block space (LADS), cervical vertigo symptoms, and function. The secondary outcome was the quality of life of patients.

### Clinical efficacy

Clinical efficacy was evaluated based on improvement of clinical symptoms and categorized as recovered, very effective, effective, and ineffective, with the following definitions: recovered: clinical symptoms disappeared and improvement rate was ≥90%; very effective: clinical symptoms and signs disappeared or significantly reduced and improvement rate was ≥75%; effective: clinical symptoms and signs reduced and improvement rate was ≥30%; ineffective: improvement rate was <30%. Improvement rate is defined as (post-treatment points - pre-treatment points) / (full score - pre-treatment points) × 100%.

### Vertigo symptoms and patient performance

The Neck Vertigo Symptom and Function Evaluation Scale by Chuhuai et al. (19) was used to evaluate vertigo symptoms and patient performance. The scale evaluates the degree of vertigo (8 points), vertigo frequency (4 points), duration (4 points), degree of neck and shoulder pain (4 points), headache (2 points), daily life and work ability (4 points), and psychological and social adaptability (4 points). Lower scores indicate less severe symptoms.

### Assessment of quality of life

The MOS-SF36 scale (20) was used to assess quality of life. The scale consists of dimensions that evaluate 1) physical health; 2) social function; 3) physical role function; 4) physical pain; 5) mental health; 6) emotional role function; 7) energy; and 8) overall health. A high score indicates better quality of life.

The patients in both groups were assessed on the first day after enrollment and after the 14th day of treatment. After six months of treatment, patients were evaluated again for the cervical vertigo symptoms, function evaluation, and MOS-SF36 scale. The number of vertigo attacks was recorded for six months.

### Statistical analysis

SPSS19.0 software (IBM, USA) was used for statistical analysis. Data are reported as means±SD. The sample size was determined using PS software (Power and Sample Size Calculation version 3.0.12, <https://biostat.app.vumc.org/wiki/Main/PowerSampleSize>). In brief, we used 0.8 mm as the mean value of the patients' LADS based on prior literature, while considering LADs <0.5 as successful recovery. Using the standard deviation of 0.51, effect size of 0.8, and P<0.05 for statistical significance, the minimal sample size was calculated to be 36. Data from before and after treatment was compared with the paired *t*-test, and between-group data was compared with the unpaired *t*-test. The chi-squared test was used to analyze categorical data.

## Results

### Patient enrollment and characteristics

**Figure 2:**
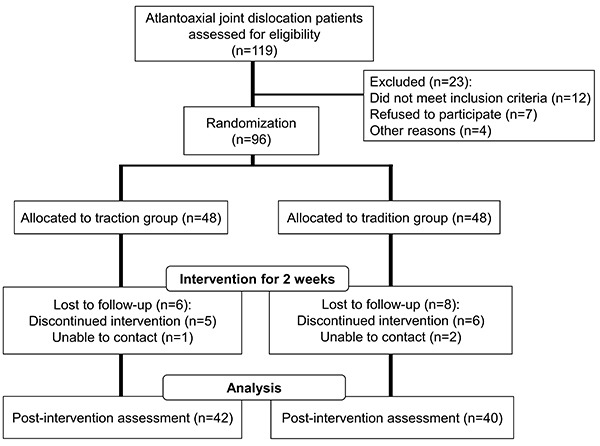
Flowchart of this study.

A total of 119 patients enrolled in the study, among which 96 were included for randomization: 48 in traction therapy group and 48 in traditional therapy group. Treatment lasted 2 weeks, and post-treatment analysis was performed based on 42 patients from the traction group and 40 patients from the tradition group, due to reasons such as loss to follow-up, discontinued intervention, or unable to contact (Figure 2). The patient characteristics of the groups are summarized in Table 1. None of the characteristics were significantly different between groups.

**Table 1:** Demographic characteristics of the included patients.

Variable	Study group		P
	Traction group (n=42)	Tradition group (n=40)	
Age (years)	39.4±10.5	41.6±11.2	0.164
Male gender, n (%)	18 (42.9%)	22 (55%)	0.377
BMI	29.4±7.2	27.9±8.6	0.181
Pre-intervention duration of disease, months	3.65±1.78	3.18±1.91	0.317
Smokers, n (%)	11 (26.2%)	13 (32.5%)	0.629
LADS, pre-intervention (mm)	0.92±0.18	0.88±0.19	0.362
Cervical vertigo symptom and function evaluation, pre-intervention			
Vertigo	8.87±2.42	8.41±2.31	0.469
Neck and shoulder pain	2.85±0.94	3.12±0.84	0.275
Headache	1.73±0.45	1.64±0.51	0.331
Activities of daily living and work scale	1.94±0.68	1.78±0.59	0.388
Psychosocial adaptation	3.33±0.86	3.47±0.95	0.294
Education			
Junior high school and below	16 (38.1%)	14 (35.0%)	0.788
Senior high school or polytechnic school	20 (47.6%)	18 (45.0%)	
College and above	6 (14.3%)	8 (20.0%)	

Data are reported as means±SD or n (%). P values for each group were derived from either unpaired *t*-test or Mann-Whitney test, as appropriate. Chi-squared test or Fisher's exact test was used for assessing distribution of observations or phenomena between groups. LADS: lateral atlantodental space; BMI: body mass index.

### Traction therapy had higher clinical efficacy than traditional therapy

After one course of treatment (14 days, 3-4 times a week), clinical efficacy was evaluated based on disappearance of clinical symptoms and improvement rate. Our results indicated that compared to the tradition group, a larger portion of patients in the traction group recovered (30.9 *vs* 22.5%) and the treatment was deemed more effective (42.9 *vs* 27.5%). Based on these data, a significantly higher clinical efficacy was found for traction therapy (P=0.038) (Table 2).

**Table 2:** Comparison of overall clinical efficacy before and after intervention.

	Study group	
	Traction group (n=42)	Tradition group (n=40)
Recovered	13 (30.9%)	6 (22.5%)
Very effective	18 (42.9%)	12 (27.5%)
Effective	9 (21.4%)	14 (30.0%)
Ineffective	2 (4.8%)	8 (20.0%)
P	**0.038**	

Data are reported as n (%). P values were derived from chi-squared test for assessing distribution of observations or phenomena between groups. Bold type indicates statistically significant.

### Traction therapy led to a significantly greater reduction in LADS

**Figure 3:**
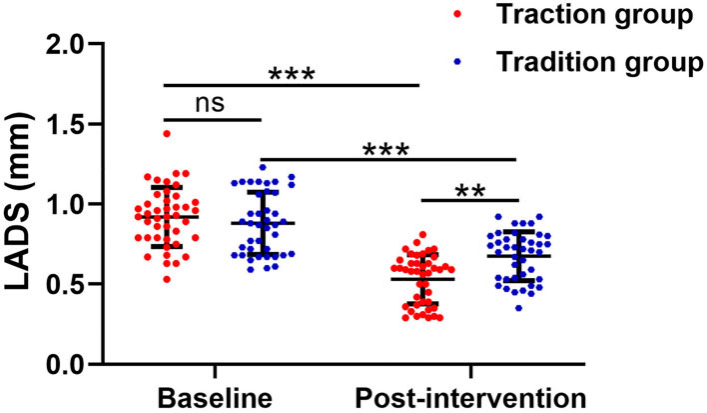
Effects of traction therapy on lateral atlantodental space (LADS) of patients with cervical vertigo due to atlantoaxial joint dislocation. Data are reported as means±SD and show all data points. **P<0.01, ***P<0.001, paired *t*-test and unpaired *t*-test. ns: not significant.

Consistent with the higher clinical efficacy of traction therapy, a greater reduction of LADS was also observed in patients who received traction therapy, compared to patients who received traditional therapy (P<0.01), although both therapies significantly reduced LADS compared to pre-treatment LADS (P<0.001) (Figure 3).

### Traction therapy had higher efficacy in alleviating vertigo symptoms and improving psychosocial function

**Figure 4:**
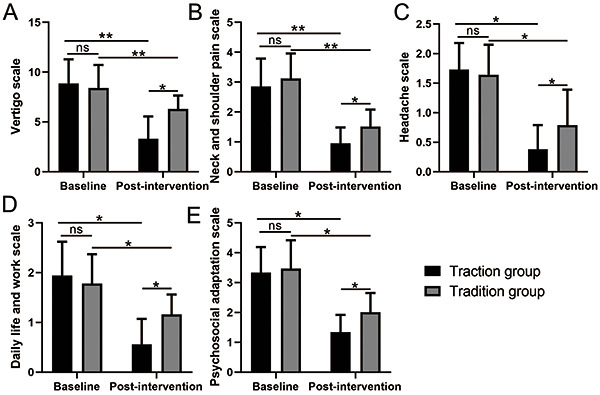
Effects of traction therapy on cervical vertigo symptoms and function (**A**-**E**) of patients with cervical vertigo due to atlantoaxial joint dislocation. Data are reported as means±SD. *P<0.05, **P<0.01, paired *t*-test and unpaired *t*-test. ns: not significant.

As shown in Figure 4, both therapies showed significant alleviation of vertigo symptoms, including vertigo (Figure 4A), neck and shoulder pain (Figure 4B), and headache (Figure 4C). Moreover, both therapies led to an increase in psychological function, reduction of impairment in activities of daily living and work activities (Figure 4D), and fewer psychosocial symptoms (Figure 4E). However, the effects of traction therapy were much stronger (P<0.05 in all variables).

### Traction therapy was more effective in improving quality of life

Overall, the traction group had greater improvement in physical function (P=0.024), role function (P=0.182), physical pain (P=0.029), general health perception (P=0.031), and social function (P=0.027) than the tradition group, which only showed significant improvement of physical function (P=0.039) and physical pain (P=0.041). (Table 3).

**Table 3:** Assessment of quality of life before and after the interventions.

MOS-SF36 (0-100)	Study group		P value
	Traction group (n=42)	Tradition group (n=40)	
Physical function			
Baseline	79.12±12.14	81.21±12.37	0.226
Post-intervention	93.24±17.5	87.33±16.82	**0.044**
P value	**0.024**	**0.039**	
Role function			
Baseline	67.65±23.18	66.15±19.79	0.241
Post-intervention	78.23±18.19	70.65±19.93	0.157
P value	0.182	0.315	
Physical pain			
Baseline	56.7±17.13	57.49±19.21	0.562
Post-intervention	80.35±19.62	70.21±16.57	**0.043**
P value	**0.029**	**0.041**	
General health perception			
Baseline	57.45±18.36	56.88±19.97	0.356
Post-intervention	75.21±17.81	63.55±16.81	0.076
P value	**0.031**	0.084	
Social function			
Baseline	71.12±17.08	72.33±19.05	0.624
Post-intervention	87.36±18.19	79.58±19.62	0.069
P value	**0.027**	0.135	
Mental function			
Baseline	66.38±19.16	65.12±18.63	0.437
Post-intervention	71.06±20.13	73.05±19.07	0.377
P value	0.172	0.243	

Data are reported as means±SD. P values were derived from paired *t*-test or Wilcoxon signed rank test, as appropriate between baseline *vs* post-intervention. P values were derived from unpaired *t*-test or Mann-Whitney test, as appropriate between groups. Bold type indicates statistically significant.

## Discussion

AJD is an important cause of cervical vertigo (21). The vertigo caused by AJD is more common in middle-aged and elderly people, but the age of onset has decreased gradually due to the increased usage of electronic devices and computers. The incidence rate is also increasing each year (3). Furthermore, treatment of atlantoaxial joint disorder can sometimes aggravate cervical vertigo, making the choice of treatment significantly important. Cervical vertigo can seriously affect activities of daily living and work, reduce quality of life, and cause great pain to patients. At present, many clinical studies are focused on the alleviation of anatomical changes rather than vertigo and quality of life. Evidence on which treatment strategies perform better in terms of reducing vertigo are lacking.

The principle of manual therapy is to restore the mechanical balance of the cervical spine and, thus, relieve vertigo. At present, manual therapy is widely used in clinics since it is easy to perform, inexpensive, and relatively safe. In this study, the benefit of traction therapy was investigated. We showed that, overall, the clinical efficacy of traction therapy was higher than that of the control group. Traction therapy also led to significantly greater improvements of cervical vertigo symptoms and function and the quality of life than the traditional therapy. The LADS, which pathoanatomically relates to the severity of AJD, also decreased, confirming that traction therapy led to a significantly higher alleviation of disease progression.

The benefits of traction therapy are attributed to an increased blood flow and reduced pain associated with the procedure (15,16). Traction therapy, which increases disk height, could result in lower internal pressure and decreased irritation of pain-sensitive fibers. The tensile stress also stretches the intervertebral disk and pulls the spine apart, and the muscles relax under the tension, making it easier to induce movement of the spinal process. In contrast, the traditional therapy to rotate the spinal process allows relatively less controllability and caries a higher risk of injuries of tissues, blood vessels, and nerves. The theoretical basis underlying the benefit of traction therapy in reducing cervical vertigo needs further investigation.

Our study is limited by the relatively small number of subjects. Also, we did not investigate the duration of the effects of therapy and long-term therapy was not implemented. We did not track the subjects' daily activities and, therefore, we cannot rule out the influence of other activities, such as exercises, on the conclusions. In addition, it is worth investigating whether our conclusions still hold when a mixed regression model is used for statistical analysis, a method considered more suitable for continuous data in randomized controlled trials and which lowers error of multiplicity. Further studies that address these limitations are warranted.

### Conclusion

This randomized controlled trial confirmed that traction therapy was superior to traditional therapy in terms of clinical efficacy, reduction of LADS, and improvement of vertigo symptoms and quality of life. The results of the study supported the clinical application of traction therapy in AJD to achieve a better outcome.

## Acknowledgments

The study was supported by the Project of Natural Science Foundation of Fujian Province (2017J0105).
